# Mr. Hughes's Letter to Dr. Jenner

**Published:** 1799-06

**Authors:** T. Hughes

**Affiliations:** Stroud


*The following Letter from Mr.* HUGHES *to Dr.* JENNER, *arriving too late for inſertion in his Work, has been tranſmitted to us; and our deſire to correct any miſrepreſentation on this ſubject, induced us to inſert it without abridgment.*

DEAR SIR,

SOME time ago, Mr. DARKE informed me, that he had communicated to you an account of the Rev. Mr. COLBORNE's family, whom he inocuţated wtth the cow-pox matter, procured by your favour. Induced by curioſity, family connecconnection, and a requeſt on the part of Mr. and Mrs. Colborne, I attended the family with Mr. Darke; and, perhaps, took a more minute account of the progreſs of the infection than Mr. Darke, at that time fully engaged, could do; and as I ſee that this matter has been miſrepreſented, I think it right that the public be furniſhed with my evidence on the ſubject, which you are at liberty to inſert in your next publication, or tranſmit to the Editors of the 'Medical and Phyſical Journal.' I underſtand that Mr. Darke omitted to mention his having inoculated his own ſervant alſo with the cow-pox matter, thinking it unneceſſary; but he has given me leave to notice this inſtance likewiſe, that no part may be omitted.

With the *cow-pox* matter were inoculated Mr. Darke's ſervant, Thomas Vick, about ſeventeen years of age, Mr. Colborne's three children, viz. Miſs Colborne, between three and four years of age, Miſs E. Colborne, between two and three, and Miſs S. Colborne, fifteen months old, and Mr. Colborne's ſervant, William King, about fifteen years of age. T. Vick, on the 13th of December, 1798, the day on which the matter was procured— Mr. Colborne's family on the following day; all by a puncture in the *left* arm.

In T. Vick there was a ſlight inflammation on the ſecond day (i.e. the day following that of inoculation), which afterwards diminiſhed, ſo that by the ſixth day hardly any appearance of the puncture remained, but a ſmall ſcab; in the evening of this day, however, a ſlight, though florid redneſs came on, accompanied by a ſmall pimple or two near the puncture; which diſappeared the next day.

In Miſs Colborne there was a diffuſed redneſs, to the extent of one fourth of an inch in diameter, with ſome hardneſs and elevation of much leſs extent, on the ſecond day. This appearance diminiſhing, was hardly perceptible by the ſixth day, and by the tenth was quite gone. She was thought to be a little heavy on the eighth day, on which pimples appeared on the face and trunk, which died away on the ninth.

In Miſs S. Colborne, there was a ſlight diffuſed inflammation on the ſecond day; the redneſs becoming more extenſive, though of a more duſky hue, the hardneſs and elevation diminiſhing to the ninth day, when the redneſs was one third of an inch diameter, and nearly covered with a thin ſcabby cruſt. On the eighth day, a few pimples were diſcerned about the face and ſcalp, which diſappeared the next day. The cruſt fell off the arm on the fourteenth, leaving a ſlight redneſs, ſucceeded by a thinner cruſt of leſs duration. A pimple appeared near the place of the puncture on the thirteenth, which withered away in two days.

Theſe

Thefe three patients were inoculated with *variolous* matter in the *right* arm; the firft on the 20th of December—Mifs Colborne and Mifs S. Colborne on the 21ft (each refpectively on the eighth day from the firft inoculation with *cow-pox* matter); and in all of them the ufual progrefs of infection from variolous matter took place, both in the appearances and fymptoms; fo that the axilla of T. Vick was affected on the fixth day from the fecond inoculation, and in the evening of the feventh the eruptive fever came on, which proved flight, he purfuing his bufinefs the whole time, and a few puftules followed. In Mifs Colborne the eruptive fever came on in the evening of the feventh, which went off on the ninth, except leaving a flight uneafinefs in the axilla, and a flight feverifhnefs for an evening or two more; and ten or twelve puftules followed. Mifs S. Colborne ailed on the tenth, and was well on the twelfth, and had three or four puftules.

The effects of the firft inoculation with *cow-pox* matter, in Mifs E. Colborne and W. King, were as follow:—In Mifs E. Colborne, the inflammation (which appeared nearly the fame on the fecond day, as in the three above related, gradually increafed, fo that by the ninth it was of the extent of half an inch in diameter. A fcab, which had been repeatedly rubbed off, probably occafioned by the part itching in the night, was renewed. She was obferved to be fretful on the eighth day, and on this day two or three pimples, like thofe of Mifs Colborne and Mifs S. Colborne, appeared, and difappeared on the ninth. By the tenth, the inflammation was of the extent of a fixpence; the vefication furrounding the fcab was about one fixth of an inch, containing matter, which feemed to be purulent. Hitherto the efflorefcence was hardly diftinguifhable from the rednefs accompanying the induration of the *fkin*; but by the fifteenth day, when the rednefs and hardnefs was of the extent of a fhilling, the margin of the efflorefcence was of the extent of a crown-piece, and of a bright colour; betwixt which and the rednefs and induration, was an interval of fkin of the natural colour. At this time, the fcab falling off, left a fore of one eighth of an inch in diameter, at firft fuperficial, but in three days more got through the fkin—the inflammation increafed. Cooling, faturnine applications, and ung. hydrarg. were applied; by which the inflammation was leffened, but not fufficiently to prevent two fmall fuppurations, a little above the fore occafioned by the puncture, one towards the infide, the other towards the outfide of the arm, each of the extent of a fhilling, and which were ready to burft on the twenty-fixth day: that on the infide burft foon afterwards, and readily healed; that on the outfide, communicating with the fore from the puncture, was kept from burfting fome days longer. On the 4th of February (the fifty-fecond day), neither fore nor induration remained. She had no illnefs, excepting the flight one mentioned on the eighth eighth day; and although the arm undoubtedly teafed her, fhe ate her food, and played with her fifters, as ufual, the whole time.

In William King, the inflammation which appeared on the fecond day, nearly as in the other patients, gradually increafed, as in Mifs E. Colborne, fo that by the 9th day it was of the extent of one eighth, and the vefication in its centre, one tenth of an inch, with three or four pimples near it. On the eleventh day, a conflux of fmall puftules uniting with the vefication, the latter by the fifteenth day was of the extent of one fixth of an inch, with a fmall fcab in its centre; its furrounding rednefs and hardnefs of the extent of a fhilling. The efflorefcence appeared on the tenth day, gradually fpread till the fifteenth, growing duller towards the centre, the margin continuing of a bright colour, when it extended one third the circumference of the arm; it had not quite difappeared on the eighteenth. On the eighth day, the axilla began to be affected with ftiffnefs, which continued to the eleventh, then abated, and on the fourteenth went quite off. On the ninth day, he had a flight head-ach. On the eighteenth, the central fcab put on the appearance of an efchar; and a day or two afterwards, the furrounding induration was of the extent of a half-crown piece; it feemed confined to the fkin, as it was moveable over the mufcles. From this time the induration leffened, and after the feparation of the efchar, which happened on the twenty-ninth, occafioning a fore, which, from the thickened ftate of the fkin, appeared of the depth of a quarter of an inch; and applying ung. hydrarg. foon went off, and the fore healed. His arm was fo little troublefome, that he went on with the ufual bufinefs of the houfe the whole time. Perhaps few, if any, practitioners would have been able, by the fight or touch, to have diftinguifhed the ftate of the arm from that occafioned by the infertion of variolous matter previoufly to the eighteenth day.

Notwithftanding we were perfuaded that the *cow-pox* matter had taken the defired effect in Mifs E. Colborne and W. King, the family being furrounded by the fmall-pox, from a general inoculation having taken place, it was thought prudent to inoculate them with *variolous matter*, in the *right* arm, at the fame time with Mifs C. and Mifs S. Colborne, viz. on the eighth day from the inoculation with *cow-pox* matter. In both, the punctures in the *right* arm inflamed earlier than they ufually do after a firft inoculation (as is common where a fecond inoculation is made fo foon after the firft, where *variolous* matter is ufed): they became fmall puftules, without any previous accompanying illnefs, without any affection of the axilla, or fubfequent puftules.

 These

Thefe two patients were inoculated a third time on the firft of January, the 12th day from the fecond, and nineteenth from the firft inoculation, with limpid *variolous* matter taken from the vefication of the right arm of Mifs Colborne, and immediately inferted. This was not followed by any inflammation, the punctures being hardly perceptible either on the fifth or eighth day.

This account of the above patients being given neither from *hearfay evidence*, nor from memory, but from notes taken at the time the obfervations were made, the reader will judge what credit is due to the *authentic information* * procured, and lately publifhed concerning them, and will make his own remarks. For my own part, I did confider at the time, and do ftill confider, the inflammation of Mifs E. Colborne's arm, as a *troublefome and difagreeable*, but not as an *alarming* and *dreadful* circumftance.

I remain, very refpectfully,
Dear Sir,
Your humble fervant,

*Stroud, 9th May*, 1799. T. Hughes."

P. S. In the above narrative it is noticed, that Mifs Colborne was thought to be a little heavy on the eighth day from the firft inoculation with cow-pox matter, and that fhe and Mifs S. Colborne, who did not take the infection of the cow-pox, as well as Mifs E. Colborne, who did, had an eruption of pimples on that day, which difappeared on the next. Nothing but the moft fcrupulous regard to mention every circumftance that occurred during the procefs of inoculation, could have induced me to infert thefe; for the heavinefs, if any, in Mifs Colborne, was very flight, and the pimples in all were fo minute, and fo tranfient, that without a different ftate of the inoculated part, they could not have been confidered by any practitioner as figns of infection having taken place, and I am perfuaded would, at any other time, have paffed unobferved."

---

In the Gloucefter Journal for May 13th, we obferved a letter on the cow-pox, figned C. Cooke, in which the following paffages occur, viz.

" Permit me to remark, that I not only *very much doubt cow-pox being a permanent preventive from fmall-pox*, but I am *confirmed* in this opinion by *occurrences* in my own practice, by converfing with many medical gentlemen upon the fubject, and by an additional account received from Dr. Beddoes, who writes (alluding to a publication edited by him), '*I have added a cafe, where fmall-pox was taken after cow-pox had been twice gone through.*' Great advantage however may arife in practice from the prefent

* Medical Communications, &c. by Dr. Beddoes, p. 401.

prefent inveftigation; becaufe knowing that cow-pox has a *temporary influence* upon fmall-pox, we can fupprefs the progrefs of it by immediately inoculating cow-pox; and could we afcertain the time to which the influence of inoculated cow-pox extends itfelf, we might always leffen the hazard of of fmall-pox recurring, by repeating the inoculation of cow-pox when neceffary.

"I will only add, that a fpurious kind of cow-pox fometimes occurs, and is an objection to the new mode of inoculation; but I *moft heartily wifh* every advantage and fatisfaction may be derived from a full inveftigation of this fubject; and remain your obliged fervant, &c."

*Gloucefter, May 9th,* 1799.

We fhall be much obliged to the writer of the letter, or any other Gentleman, for an account of any occurrences which tend to confirm his opinion, as we have never been able to find one unequivocal cafe of fmall-pox following the true cow-pox, though we know many inftances where all probable means were ufed to infect the patients with fmall-pox, and this at very different intervals after their having undergone the cow-pox.

